# A pragmatic evaluation of a public health knowledge broker mentoring education program: a convergent mixed methods study

**DOI:** 10.1186/s43058-022-00267-5

**Published:** 2022-02-15

**Authors:** Emily C Clark, Bandna Dhaliwal, Donna Ciliska, Sarah E Neil-Sztramko, Marla Steinberg, Maureen Dobbins

**Affiliations:** 1 National Collaborating Centre for Methods and Tools, McMaster University, McMaster Innovation Park, 175 Longwood Rd S, Suite 210a, Hamilton, ON L8P 0A1 Canada; 2 School of Nursing, McMaster University, Health Sciences Centre, 2 J20, 1280 Main St W, Hamilton, ON L8S 4 K1 Canada; 3 Department of Health Research Methods, Evidence & Impact, McMaster University, McMaster University Medical Centre, 2C Area, 1280 Main St W, Hamilton, ON L8S 4 K1 Canada; 4 Vancouver, Canada

**Keywords:** Evidence-informed decision-making, Knowledge broker, Public health, Knowledge translation, Research evidence, Professional development, Capacity building, Organizational change, Mixed methods

## Abstract

**Background:**

Public health professionals are expected to use the best available research and contextual evidence to inform decision-making. The National Collaborating Centre for Methods and Tools developed, implemented, and evaluated a Knowledge Broker mentoring program aimed at facilitating organization-wide evidence-informed decision-making in ten public health units in Ontario, Canada. The purpose of this study was to pragmatically assess the impact of the program.

**Methods:**

A convergent mixed methods design was used to interpret quantitative results in the context of the qualitative findings. A goal-setting exercise was conducted with senior leadership in each organization prior to implementing the program. Achievement of goals was quantified through deductive coding of post-program interviews with participants and management. Interviews analyzed inductively to qualitatively explain progress toward identified goals and identify key factors related to implementation of EIDM within the organization.

**Results:**

Organizations met their goals for evidence use to varying degrees. The key themes identified that support an organizational shift to EIDM include definitive plans for participants to share knowledge during and after program completion, embedding evidence into decision-making processes, and supportive leadership with organizational investment of time and resources. The location, setting, or size of health units was not associated with attainment of EIDM goals; small, rural health units were not at a disadvantage compared to larger, urban health units.

**Conclusions:**

The Knowledge Broker mentoring program allowed participants to share their learning and support change at their health units. When paired with organizational supports such as supportive leadership and resource investment, this program holds promise as an innovative knowledge translation strategy for organization wide EIDM among public health organizations.

**Supplementary Information:**

The online version contains supplementary material available at 10.1186/s43058-022-00267-5.

Contributions to the literature

- This study implemented and evaluated an intensive Knowledge Broker mentoring program to facilitate evidence informed decision-making, using an innovative mixed methods design for deeper analysis than is possible with quantitative methods alone.
- The findings highlight that the key factors for program success are integration of knowledge brokers into activities across the organization, embedding evidence into decision-making processes, and supportive leadership with organizational investment of time and resources.
- These findings contribute to the broader literature for successful implementation of knowledge broker roles to facilitate evidence-informed decision-making, and notably explore successful implementation in smaller, rural organizations, where limited success has been achieved.

## Introduction

Evidence-informed decision-making (EIDM) in public health integrates the best available evidence from research and the local context to optimize decision-making. Achieving EIDM requires, in part, finding, synthesizing, and applying the best available evidence from research sources, community data and local contexts, societal and political preferences, and available resources [1–3]. EIDM can help maximize population health outcomes given finite public health resources by implementing strategies with known effectiveness [4, 5]. The Public Health Agency of Canada outlines core competencies for the public health workforce including finding, analyzing, and applying evidence from research and community sources [6]. In Ontario, Canada, EIDM has been identified as a foundational standard for practice [2].

Despite these expectations, barriers to achieving EIDM persist. Public health practitioners do not universally have the knowledge and skills for finding, appraising, and using evidence in practice; some public health organizations are unable or unwilling to support evidence-informed practice [7–9]. Strategies that focus solely on building individuals’ skills for EIDM have been effective in increasing the practitioners’ capacity but have not had organization-wide impact [10–12]. To achieve an organizational shift to EIDM, strategies must facilitate organizational change whilst building individual capacity [5, 13, 14]. In this context, the term “capacity” refers to the knowledge, skill, and ability to apply EIDM in practice, reflecting both competence and situational support [5, 15, 16].

Reviews of interventions to increase research use in public health have found that strategies should be adapted to the needs and priorities of the organization and individuals [17–19]. Knowledge broker (KB) roles are inherently well-suited to meet this requirement, as the scope of a KB’s role is linked to the organizational context [20]. KBs are responsible for knowledge management (obtaining relevant evidence, creating tailored knowledge products, supporting evidence sharing), knowledge linkage and exchange (facilitating collaboration, developing and maintaining networks), and capacity building (helping develop analytic skills, facilitating and evaluating change) [20]. In Canada, KBs have long been a component of efforts to support EIDM [21]. KBs in Canada are found across diverse contexts, such as facilitating stakeholder relationships for income assistance policies for people who use drugs [22], bridging the research-practice gap for uptake of measurement tools in children’s rehabilitation clinics [23], and partnering with secondary schools to facilitate the uptake of interventions to promote and improve student health [24]. While results of studies evaluating KB interventions in public health settings are mixed, KBs show promise in supporting organization wide EIDM [19, 25–32].

This pragmatic study sought to determine the effect of an intensive KB mentoring program delivered by the National Collaborating Centre for Methods and Tools (NCCMT) to facilitate organizational change for EIDM including organizational level use of evidence, perceptions of the value of the KB mentoring program, and public health units’ success or challenges in integrating EIDM.

## Methods

### Study design

A convergent mixed methods design was used to evaluate two sequential cohorts of the KB mentoring program, with individuals from five public health units participating in each cohort. In the quantitative phase of the study, senior leadership at each health unit participated in a focus group to set goals for research use. Following program completion interviews with participants and their managers were deductively coded to quantify the extent to which the goals were achieved. In the next phase, a descriptive qualitative study [33] investigated why some participating health units were more or less successful in implementing EIDM through inductive analysis of in-depth, semi-structured interviews. Findings from the quantitative and qualitative phase were merged and used to evaluate the overall impact of the KB mentoring program, as well as how and why health units achieved their identified goals. The study is reported following the Good Reporting of a Mixed Methods Study (GRAMMS) checklist [34]. Ethics approval was obtained (Hamilton Integrated Research Ethics Board Project #15-016), and participants provided written informed consent.

### Sample

#### Organizations

Any Canadian public health organization involved in front-line service delivery was eligible to participate in the KB mentoring program. The NCCMT issued an open call through its newsletter and contacted organizations with previously expressed interest in the program. As a result of these efforts, ten public health organizations confirmed their participation in the program over two sequential cohorts. The first cohort ran from January 2015 to December 2016, and the second cohort ran from January 2017 to June 2018.

#### Individuals

Organizations identified five-to-six staff members to participate in the program by seeking volunteers or strategic selection. Diverse staff positions were eligible to participate including public health nurses, health promoters, dietitians, public health inspectors, librarians, managers, or others.

### Knowledge broker mentoring program

The program’s objectives were to assess and assist public health organizations to develop organizational capacity for EIDM, as well as to build capacity among selected staff to function as “internal” knowledge brokers in evidence-informed practice. The program is described according to the TIDieR (Template for Intervention Description and Replication) Checklist; a completed TIDieR Checklist is included in Additional file 1: Appendix 1 [35]. Participants enrolled in the program were given opportunities to develop their knowledge about the EIDM process including the types of evidence used in public health decision-making and skills related to searching for, appraising, synthesizing, and applying evidence to practice. The program, co-designed with public health professionals, was also informed by collective decades of experience delivering EIDM education to public health. The four NCCMT knowledge translation specialist mentors who delivered the program held relevant graduate degrees and a wealth of experience in public health and EIDM. Mentors were assigned 1–3 organizations in the first cohort and two organizations in the second cohort. Intervention content was identical for each cohort.

The program began with an organizational assessment with the senior leadership team of each health unit. Assessments identified the leadership team’s EIDM goals for the program specifically and the organization generally.

Participants took part in a 5-day in-person workshop, followed by a 3-day workshop at 6 months, and a 2-day workshop at 12 months held at McMaster University in Hamilton, ON. A printed course pack containing the course syllabus and copies of the required readings were given to participants prior to the start of the Program. Workshops were held daily from 9 AM to 4 PM, consisting of a small number of didactic lectures, with most time spent in small-group problem-based sessions; content focused on a systematic approach to EIDM [1]. Between workshops, participants convened virtually each month to share progress and continue practice-based learning through group critical appraisals of research evidence. Groups at each public health organization met with mentors every 2–4 weeks for a 30-to-60-min consultation via teleconference to address emerging questions and strategize organizational change for EIDM. During the final 6 months of the program, participants completed a rapid review [36]. Participants selected synthesis topics with senior management to ensure findings would benefit the public health organization. Completion of these rapid reviews reinforced the EIDM skills participants gained during the program, including defining an answerable research question, searching for literature, critically appraising included studies, and synthesizing results for a final report. Examples of topics include workplace interventions for mental health, community interventions for testing and treatment of chlamydia, and characteristics of natural environments that affect youth mental health and wellbeing. A full list of research questions is included in Table 1.

**Table 1:** Characteristics of participating health units

Area (km^2^)	No. of health units
1000–5000 km^2^	8
5000–10,000 km^2^	1
10,000–15,000 km^2^	1
Size of Population Served (persons)	
< 50,000	1
50,000–100,000	3
100,001–500,000	2
> 500,000	4
Population density (persons/km^2^)	
1–50	2
50–100	2
100+	6
Population type	
Large immigrant population	3
Mostly non-immigrant	7
Urban/rural	
Urban and rural	5
Urban, large urban core	1
Mostly rural	3
Rural, northern	1
Main industry	
Health care and social assistance	5
Manufacturing	3
Public administration	1
Retail	1
Number of employees	
< 150	4
100–400	5
> 400	1
Board of health structure^a^	
Single-tier	1
Regional	2
Autonomous	5
Semi-autonomous	1
Autonomous/integrated	1
Rapid review research questions	
• What are effective workplace interventions to reduce anxiety and work stress in an office setting? • Which characteristics of natural environments are most impactful for mental health and wellbeing among adolescents (12 to 17 years) and young adults (18 to 24 years)? • Is the use of social media effective at promoting healthy lifestyles and reducing weights among individuals 13 years or older? • What are the effective psychological or psychosocial interventions to prevent diagnosed perinatal mood disorders? • What are factors that impact an individual’s preparedness for emergency? • Among youth aged 13–25 years, which interventions have the greatest impact on reducing teenage pregnancy rates? • What community interventions are effective to increase uptake and adherence of testing and treatment of chlamydia in males aged 20–29? • How do celebrities’ actions impact the health-related behavior, knowledge, and attitudes of individuals or groups of individuals? • Are interventions effective in promoting smoking cessation and, if yes, which ones? • What are the effective built environment strategies that a health department could undertake to promote positive mental health and wellbeing in children and youth in our city?	

^a^Most units operated as autonomous structures, governed separately from their municipalities while three were integrated with municipalities, meaning they operated within their municipalities’ administrative structures and reported to city management

### Phase 1: Quantitative study component

Characteristics of each participating health unit and the populations they serve were collected from government websites [37]. Participant demographic questionnaires were completed at baseline. Descriptive statistics were used to summarize participant demographics.

Organizational goals for EIDM and program participation were captured using the validated organizational self-assessment tool *Is Research Working for You?* [38]. Implementation of EIDM was assessed through telephone interviews between 6 and 18 months following program completion. The third-party interviewer had extensive experience in qualitative evaluations in public health, leadership development, and knowledge translation. All interviews were recorded and transcribed verbatim with identifying information removed. Data management and coding was facilitated with the use of NVivo 12 Plus. Interviewees included a purposeful sample of program participants, managers, and senior decision makers to obtain perspectives from participants and non-participants at various levels of each health unit.

Interviews probed the program’s organizational impact in terms of overall staff capacity and system and process changes toward realizing the organization’s EIDM goals. Interview questions are included in Additional file 2: Appendix 2. Goals set by each participating public health organization during organizational assessments at the program start were used to deductively code interviews [39]. In quantifying evidence for having met goals, there was substantial evidence for achieving a goal if the goal condition was described by at least three interviewees, including at least one manager; and there was some evidence if the goal condition was described by some interviewees but not a manager.

### Phase 2: Qualitative study component

Following the quantification of goals achieved by each health unit, a descriptive qualitative study [33] was conducted to identify how EIDM was implemented within each health unit. Descriptive qualitative studies explore phenomena or processes from the perspectives of those involved, and findings can often be integrated in a mixed methods analysis [33]. The qualitative component of this study allowed for insight into why some participating health units were more or less successful in reaching goals for implementing EIDM.

Data were also analyzed using a conventional content analysis approach to describe processes related to EIDM implementation [40]. Categories that emerged from this conventional analysis were organized into meaningful clusters [41].

### Phase 3: Data integration

Quantitative and qualitative data were merged in a mixed methods data analysis [42, 43]. Health units’ overall success in EIDM implementation was measured through quantitative analysis of evidence for meeting goals, and their successes were explained through integration of results of an inductive analysis of interviews. Specifically, categories identified in the qualitative analysis were linked to each organization’s stated goals, such that categories provided insight into factors that impacted EIDM implementation.

## Results

### Participant characteristics

#### Public health organizations

Ten public health units in the province of Ontario, Canada, participated in two cohorts of the program. Participating health units served diverse populations and geographies (Table 1). Two health units invited staff to volunteer, five selected staff to participate, and three used a combination of these approaches.

#### Individuals

**Fig. 1:**
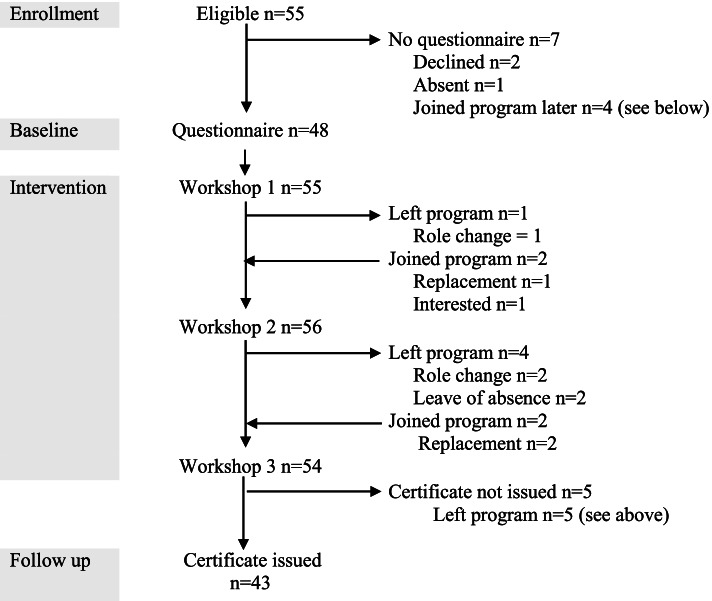
Participant flow diagram

Fifty-five participants took part in the program, ranging from four to eight participants per public health unit. Five participants left the program due to role changes within their public health unit or leaves of absence and were replaced by new participants. Demographic data were collected from 51 participants (Table 2). Two participants declined to complete the demographic questionnaire, and one was absent when questionnaires were completed. One participant was added to the program following the first workshop based on interest in the program (Fig. 1).

**Table 2:** Participant demographics

	Cohort 1 *n* (%)	Cohort 2 *n* (%)	Combined *N* (%)
Total	27	24	51
**Gender**			
Female	24 (88.9)	21 (87.5)	45 (88.2)
Male	3 (11.1)	3 (12.5)	6 (11.8)
**Years working in public health** (mean ± SD)	10.4 ± 8.3	9.3 ± 5.8	9.9 ± 7.2
**Education level**			
Bachelors	14 (51.9)	13 (54.2)	27 (52.9)
Masters	11 (40.7)	11 (45.8)	22 (43.1)
Doctorate	2 (7.4)	0	2 (3.9)
**Main job title**			
Communications consultant	0	1 (4.2)	1 (2.0)
Dental hygienist	0	1 (4.2)	1 (2.0)
Director	1 (3.7)	0	1 (2.0)
Epidemiologist	1 (3.7)	2 (8.3)	3 (5.9)
Librarian	1 (3.7)	1 (4.2)	2 (3.9)
Manager	1 (3.7)	1 (4.2)	2 (3.9)
Nurse practitioner	0	1 (4.2)	1 (2.0)
Nutritionist or dietician	3 (11.1)	1 (4.2)	1 (2.0)
Program or project coordinator	3 (11.1)	2 (8.3)	5 (9.8)
Public health inspector	4 (14.8)	5 (20.8)	9 (17.6)
Public health nurse	9 (33.3)	7 (29.2)	16 (31.4)
Other			
**Division**	4 (14.8)	2 (8.3)	6 (11.7)
Administration	6 (22.2)	8 (33.3)	14 (27.5)
Environmental health	6 (22.2)	5 (20.8)	11 (21.6)
Infant and child development	4 (14.8)	1 (4.2)	5 (9.8)
Public health nursing and nutrition	11 (40.7)	9 (37.5)	20 (39.2)
Oral health	0	1 (4.2)	1 (2.0)

### Organizational change for EIDM

Organizational goals for EIDM were grouped into themes, with specific priorities per organization identified in Table 3. One public health unit has been omitted from this analysis after not providing post-program data. Levels of success in reaching identified EIDM goals varied (Table 3). In some cases, there was a disconnect between interviewees perceptions at the same public health unit. For example, managers reported that staff were allocated sufficient protected time for EIDM work, while staff noted they needed more time.

**Table 3:** Achievement of EIDM goals by health units

Category	Goal	Cohort 1				Cohort 2				
		1^a^	2	3	4	6	7	8	9	10
Increase the organization’s capacity for EIDM	Increase staff skills for EIDM	++	++	++	++	+	++	−	++	++
	Increase resources for EIDM	+	n/a	+	++	n/a	n/a	n/a	n/a	++
	Increase time for EIDM	++	−	+	n/a	n/a	+	−	+	+
	Increase research acquisition	n/a	−	n/a	n/a	n/a	n/a	−	n/a	n/a
Integrate research evidence use into processes	Use research in work more often	++	+	+	++	++	++	+	−	++
	Assess and adapt research to local context more often	++	+	+	+	−	n/a	−	−	n/a
	Systematically integrate research evidence	n/a	n/a	n/a	n/a	++	n/a	n/a	n/a	n/a
	Use research consistently in decision-making	++	+	+	+	n/a	++	−	−	+
	Consider the quality of evidence when making decisions	+	n/a	n/a	n/a	n/a	+	n/a	n/a	n/a
	Directors hold management accountable for using evidence	n/a	+	n/a	n/a	+	n/a	n/a	n/a	n/a
Develop a culture for EIDM	Increase priority of using research	n/a	+	++	n/a	n/a	++	n/a	++	++
	Acceptance of time used for EIDM learning	++	n/a	n/a	++	n/a	++	n/a	n/a	n/a
	Acceptance of time used for EIDM practice	−	n/a	n/a	++	n/a	+	n/a	−	n/a
	Work with external partners	++	−	++	n/a	−	++	−	n/a	++
	Learn from peers within health unit	++	n/a	++	n/a	++	n/a	++	n/a	n/a
	Incentivize staff to use EIDM	n/a	n/a	n/a	n/a	−	n/a	−	n/a	n/a

^a^Health units were assigned numbers for anonymity

++ Substantial evidence for achieving goal

+ Some evidence for achieving goal

− No evidence for achieving goal

n/a Not identified as goal

Further inductive analysis of interviews explored factors and themes that may have contributed to public health units’ success and challenges in integrating EIDM.

#### Increased organizational capacity for EIDM

Each health unit prioritized improving staff skills for EIDM and using research more often. Most prioritized providing staff with time and resources for EIDM. While most reported staff had increased their skills for EIDM, some indicated there was insufficient time and resources to support applying EIDM. Findings reveal strategies for increasing skills, including participants stepping into EIDM support or teaching roles and training additional staff. Reasons why EIDM capacity goals were not achieved included limitations of the program and participants’ transition out of current roles.

#### Participants consulting or teaching peers

In seven health units, participants entered teaching roles, in some cases, acting as informal consultants within teams or departments. An executive noted that participants, “have, to a certain extent, become champions [of EIDM] within their teams” (Health Unit 7, Senior Executive Z). Some participants were formally assigned with providing orientation and guidance for EIDM. Other participants became informal sources of knowledge and guidance for colleagues, “…[participant] walked our staff through how to apply this approach and so as a result, a brief review was done of the intervention and literature related to it.” (Health Unit 6, Manager V).

At four health units, participants provided formal workshops to the broader organization. One health unit had participants deliver a series of presentations on EIDM, while three others implemented monthly journal clubs where participants led appraisals of journal articles with colleagues.

Overall, management and peers recognized program participants as resources for EIDM. Various strategies allowed participants to share their expertise, reinforcing participants’ learning and developing other staff’s skills.

#### Limitations of program

Some participants felt the program curriculum did not directly or easily transfer to their work. A participant noted little room to apply EIDM, “our programs are very clearly scripted about what we have to do, and how and why.” (Health Unit 3, Participant O).

Others noted that the program’s focus on research evidence did not equip them for applying other types of evidence,

“When you look at the model about [EIDM] we focused a lot on the research bubble. And so things like the political climate and resources, those other bubbles that are in that model weren’t really touched on but yet I know from my own job that they’re extremely important.” (Health Unit 2, Participant G)

These criticisms of the program curriculum related to its applicability to participants’ work and whether the program adequately prepared participants to apply EIDM in different contexts.

#### Participant transitions to new roles

Most health units encountered staff turnover during the program. At one health unit, half of the participants had transitioned to new roles by the time the interviews were conducted. It was noted that participants were typically high achievers, which led to their promotion to new roles, “what happened was probably partly because the individuals we selected were high performers and were leaders in and of their own right, but over time they went on to other positions” (Health Unit 1, Manager A). Other participants left roles for personal reasons. Loss of these participants hindered implementation of EIDM, especially as they had become champions for EIDM.

### Integration of EIDM into processes

Health units prioritized using evidence more consistently in decision-making. Most sought to assess and adapt research evidence more often while fewer prioritized considering quality of evidence, systematically integrating evidence use and holding management accountable for research use.

Thematic analysis uncovered that some health units concretely integrated EIDM into decision-making processes by developing structures, processes or templates to support EIDM, creating or adapting staff positions for EIDM and pursuing additional learning opportunities.

#### Structures, processes, or templates to support EIDM

Six health units described embedding EIDM into reporting templates or processes to prompt providing an evidence base for program planning recommendations. One health unit revised its briefing note template, “We’ve got a template and a process for doing briefing notes [that includes] what level of literature research are you using” (Health Unit 1, Manager B). Some health units integrated resources supporting EIDM, such as guidelines and tools, into various processes. Others described guides or resource hubs to support all staff at the organization to engage in EIDM. A resource library was shared through a health unit’s internal network to help staff apply EIDM in program planning. These various strategies helped reinforce and remind staff to use EIDM in all processes.

#### Staff positions dedicated to EIDM

Three health units noted that roles had changed to integrate EIDM work, “because with the training we were given dedicated time, dedicated FTE [full-time equivalent], all of us, and I think the dedicated FTE really helped us” (Health Unit 4, Participant R).

At two health units, additional staff were hired in specialist roles for EIDM support. On the growing number of research analysts at a health unit, “when I first started, I was one of the first [research analysts] and there were only about three at the time, and now I think there's four or five of them” (Health Unit 4, Participant Q). A dedicated specialist role had been created and filled at another health unit, to further educate frontline staff and guide them through rapid review processes.

Staff noted that dedicated time and roles to EIDM not only facilitated implementation, but it reinforced the prioritization of EIDM at the organization.

#### Lack of plan for participants

Five health units reported specific goals that had not been achieved. Issues included lack of direction for participants,

“We really had to … remind people that we have this training. It wasn’t necessarily, okay, you guys have this training now, we’re going to get you to do this, this and this or this is, kind of, the plan to have it sustainable. It’s, kind of, been us that’s been advocating for the sustainability of it.” (Health Unit 2, Participant H)

Plans for participants at these health units were absent. Newly acquired knowledge and skills were not applied or shared more broadly within the health unit, which led to frustration from participants.

#### Lack of protected time

Interviewees at six public health units expressed frustration at not dedicating enough time for EIDM practices. Noting that it was difficult to balance staff time to meet requirements while also using evidence,

“I think the challenge is always to sort of protect the time of those people so that they’re able to act as sort of a knowledge broker for the team and that they’re not struggling to sort of meet that need while continuing to do 100 percent of their normal job.” (Health Unit 10, Manager PP)

Interviewees noted that decision makers needed to allocate time dedicated to EIDM work to ensure that EIDM remained a priority and the work sustainable. Similar to lacking a concrete plan for participants to apply their EIDM skills, participants were frustrated at not having the opportunity to apply the knowledge and skills gained from the program.

#### Changes at the health unit

Some health units faced other factors that limited their focus on EIDM. Three had unexpected large-scale changes, such as in leadership, organizational structure or a merger with another health unit. Changes significantly overshadowed EIDM as a priority, “We’ve had a lot of change in upper leadership… and just because of all that flux and change we haven't really had a chance to really dig in [to integrating EIDM]” (Health Unit 2, Participant I). Similarly, merging with another health unit delayed progress with EIDM,

“We released our rapid review just as the merger was happening and, you know, we were without phones and fax machines and computers, and the knowledge broker piece was very much lost in that shuffle, and it's just picked up momentum again.” (Health Unit 9, Participant LL).

While the changes described by interviewees were varied, the effect was consistently a lack of focus on EIDM and lower prioritization of EIDM implementation.

### Culture

Fewer goals were set for staff attitudes and organizational culture. Many health units prioritized expanding work with external partners. Health units commented on varying extents of culture shift. Culture changes included leadership support for EIDM, expectations that decisions would use EIDM, acceptance of time for learning and doing EIDM, and peer learning.

#### Leadership support

Six public heath units’ leadership set expectations for the transition to an EIDM approach. Actions of Medical Officers of Health were specifically cited, “EIDM and the use of the knowledge brokers within the office of the Medical Officer of Health [supported] ongoing involvement and leadership from the MOH office in creating a culture of [EIDM]” (Health Unit 3, Senior Executive K). Noted of another Medical Officer of Health,

“She’s always wanting, you know, to know like where does this come from, you know, and it’s not to kind of, you know, be testing you or whatnot, but it’s really wanting to be grounded in the evidence and that like all the – everything that we do, our practice is to be grounded in evidence.” (Health Unit 7, Manager AA)

Leadership was described as especially impactful for helping generate organization-wide interest in EIDM and an appreciation of its value, beyond participants and their teams.

#### Expectations for using EIDM

Some health units described a profound shift in expectations for EIDM. Compared to before the program, “I find that staff are more apt to say ‘well, we should look at the research.” (Health Unit 10, Manager OO). At another public health unit, “[EIDM} is definitely the status quo now. It's present everywhere” (Health Unit 4, Participant Q).

At another health unit, not only is there an expectation to use evidence, but to use evidence appropriately, “before, staff would say, ‘We really want to do this, here’s the evidence to support it, let's go’, and we’re able to say, ‘Actually I think that came in the wrong order, what's our question, let's find the evidence and then let's make a decision’” (Health Unit 4, Participant T).

Overall, these health units described environments where applying EIDM had become an expectation. Staff had become accustomed to preparing evidence and managers consistently required evidence to support decision-making.

#### EIDM across public health roles

Participants represented a variety of core public health roles, including typically office-based such as policy analysts and community-based positions, such as public health inspectors (Table 2). Participants in diverse roles described contributing to EIDM implementation. For example, a public health inspector described:

“I think another big way that it’s also impacted me is in my work as a health inspector. I would say … I’ve definitely increased in our peer review literature that I’m looking at and also working with some of the committees that I work on here to disseminate that to the health inspectors.” (Health Unit 7, Participant DD)

Participants who worked in the community were not limited in applying EIDM compared to participants who worked in offices with more consistent computer access. Participating public health inspectors in particular described championing EIDM among their teams.

#### Acceptance of time for EIDM

Three public health units allocated time specifically for staff to develop skills for EIDM, and managers were accepting and encouraging of this practice.

“People are just really taking the time [for EIDM] and, supervisors and, managers are providing the time. They're very supportive of giving employees time to learn about this, which is a shift in itself.” (Health Unit 7, Participant BB)

While a lack of protected time to apply EIDM hindered implementation, the provision of time for staff to develop EIDM skills was described as valuable. Acceptance of this time allowed participants and other staff to develop their skills without time pressures.

#### Lack of staff buy-in

Four public health units found staff on teams that did not have a participant from the KB mentoring program embedded in the team were reluctant to change their processes. For some, this was attributed to the time required for EIDM.

“… there probably still is the tendency for some of the areas to want to find the quick answer … they would be more likely to just search for something that supports [their opinion] as opposed to taking the time to ask it in a more objective way.” (Health Unit 3, Participant M)

Reluctance to adopt EIDM in some departments was due to biases, specifically to participants’ perceived lack of career experience,

“But my team – I had people on my team going ‘I’m not listening to her. She’s only been here for like three years!’ … she does have all this great knowledge, but it just doesn’t work that way.” (Health Unit 10, Manager OO)

In some cases, staff reluctance was directly related to EIDM processes, or to external factors, such as interpersonal relationships.

#### Lack of EIDM understanding among management

Some participants’ immediate supervisors did not appreciate how EIDM would affect day-to-day work and did not set appropriate expectations. Training of management would be beneficial,

“… engaging a few more at the management level would have resulted in perhaps a few of those glitches that we experienced, like mitigating those a little bit in terms of how we supported more of our frontline staff … and how to best support the team in terms of sharing their learning and building on it more systematically than we were.” (Health Unit 4, Manager P)

There was frustration that workloads and time were not managed effectively. While EIDM work was added, there were no other projects removed from portfolios, leading to participants becoming overwhelmed.

### Summary of themes for organizational implementation of EIDM

Interviews provided insight into the successes and challenges faced by program participants in implementing EIDM. Thematic analysis uncovered several major factors for the implementation of EIDM on an organizational level, including building capacity for EIDM, integrating EIDM into processes, and organizational culture.

## Discussion

Over two cohorts, ten public health units in Ontario participated in an intensive KB mentoring program. Public health units reported varying degrees in meeting their identified EIDM goals. These results show the real-world attainment of organizational EIDM goals through such a program and the factors that influenced EIDM implementation.

Achievement of EIDM goals varied across organizations. Interviewees at some organizations reported culture shifts to widespread adoption of EIDM; others saw limited implementation. Organizations that demonstrated readiness through strong leadership supportive of resource investment and a culture accepting and enthusiastic for change achieved greater success in realizing EIDM goals. Similar readiness factors have been shown to be important factors for EIDM [5, 13, 19, 26, 28, 44]. This study’s findings illustrated that leaders who established clear expectations for EIDM from staff also helped facilitate resource investments, such as dedicated EIDM support staff and clear plans for program participants to champion EIDM within the organization. These findings are consistent with previous studies in public health settings, where supportive leadership, organizational readiness, and integration of EIDM processes predicted success [19, 26, 45, 46].

Smaller, rural public health units were successful in implementing EIDM, demonstrating that size and location of organizations do not limit EIDM practices. Several of the smallest and most remote participating organizations were successful in achieving their EIDM goals, despite their own expectations of limited success due to relatively fewer available resources. As noted by participants, the impact of program participants was perceived to be greater in smaller organizations than in larger ones. Previous EIDM implementation studies in health-related rural and remote settings identified perceived barriers to EIDM, such as gaps in knowledge, unfavorable attitudes, and lack of resources [47–51]. This study’s findings provide an alternative perspective to these barriers and may enable smaller, rural organizations to overcome perceived barriers to EIDM.

Participants’ professional roles did not limit contributions to EIDM practices, consistent with findings reported by others [26, 45]. This study found that public health inspectors contributed to advancing EIDM to similar extents as colleagues in other professional roles that are based in offices for their work rather than mobile in the community. Front-line environmental health staff have previously reported tension between mandated practices and emerging evidence, lack of evidence for emerging topics, perception that only medical research evidence should be used and challenges in measuring environmental health-related outcomes [52]. In this study, public health inspectors overcame many previously identified barriers for implementing EIDM, suggesting a variety of public health roles can engage in EIDM. Others have noted the important role managers play in supporting public health inspectors to engage in EIDM [52].

One limitation of the KB mentoring program was the focus on using research evidence, rather than data from other sources such as local context. Local evidence has been previously found to be more influential than external evidence [30]. Further training on the EIDM steps of implementation and evaluation were also identified. This is consistent with reports of EIDM training in public health, noting that staff value evidence from local contexts but lack skills to effectively gather, appraise, and synthesize other forms of evidence, and that evaluation is not well-supported [7]. To integrate participate feedback, future program iterations should provide a broader curriculum, including finding and assessing non-research evidence, synthesizing multiple types of evidence, adapting evidence to local contexts, and developing and evaluating implementation plans.

The study did not include a control group and as such it is not possible to distinguish the program’s effects from changes that may have occurred without program participation. In future iterations, a time series or stepped wedge design, where organizations are observed prior to intervention, may help further determine the program’s specific effects [53].

KB roles can pose challenges, both for the individuals acting as KBs and the organizations they serve. The KB role is not well-defined, so contributions vary greatly in different contexts [20, 54]. Since KBs function between the worlds of evidence and practice, they may not be considered experts or accepted by colleagues in either community [55]. To minimize exclusion by public health colleagues, this program built capacity of staff already practicing and experienced within public health. In some cases, more junior participants’ attempts to share knowledge were dismissed by senior staff. It is important to carefully consider how staff chosen to participate in such programs will be accepted by peers [26]. The use of dedicated KBs may limit organizational capacity for knowledge translation, as evidence bottlenecks can form at the KB level [55]. This study found that participants transferred newly acquired knowledge and skills to colleagues. Evidence bottlenecks may be avoided with multiple people within the same organization in the role [48].

While this study offers new insights into a KB mentoring program to advance EIDM in public health, there are aspects to explore further. Enhanced focus on non-research evidence and implementation should be included in future program iterations. Participation by smaller, rural, or remote organizations should be considered to further explore EIDM implementation in settings typically perceived as lacking capacity. Exploration of EIDM implementation for diverse public health roles will optimize approaches for organization wide EIDM.

## Conclusions

This KB mentoring program was promising at an individual level, indicated by reported practice changes by program participants. Shifting organizational culture to EIDM is complex, but this program shows potential for effective integration of EIDM within organizations. A KB mentoring program can support organizations to initiate and progress toward identified EIDM goals. Important organizational factors that are key to progress toward EIDM goals include supportive leadership and invested resources.

## Supplementary Information

**Additional file 1: Appendix 1.** The TIDieR (Template for Intervention Description and Replication) checklist.

**Additional file 2: Appendix 2.** Interview structure.

## Data Availability

The datasets used and/or analyzed during the current study are available from the corresponding author on reasonable request.

## Abbreviations

EIDM: Evidence-informed decision-making
KB: Knowledge broker
NCCMT: National Collaborating Centre for Methods and Tools
