# Supplementary material for: A pragmatic evaluation of a public health knowledge broker mentoring education program: a convergent mixed methods study
Source: Implement Sci Commun. 2022 Feb 15;3:18. doi: 10.1186/s43058-022-00267-5 (PMC8845284; doi:10.1186/s43058-022-00267-5)
Supplement: Supplementary file 1 — Additional file 1: Appendix 1. The TIDieR (Template for Intervention Description and Replication) checklist. [file 43058_2022_267_MOESM1_ESM.docx]

| Item number | Item | Where located | |
| --- | --- | --- | --- |
|  |  | Primary paper  (page number) | Other (details) |
|  | **Brief name**  Knowledge Broker mentoring program, National Collaborating Centre for Methods and Tools (NCCMT) | 6 | [nccmt.ca/training/knowledge-broker-mentoring-program](https://www.nccmt.ca/training/knowledge-broker-mentoring-program) |
|  | **Why**  Assess and assist public health’s organizations to develop organizational capacity for EIDM, as well as to build capacity among selected staff to function as “internal” knowledge brokers in evidence-informed practice. | 6 |  |
|  | **What \| Materials**  Participants were given a printed course pack which included a syllabus and copies of required readings. | 7 |  |
|  | **What \| Procedures**   - Workshop participants took part in a series of didactic lectures, followed by problem-based discussions. Upon completion of the first workshop series, participants met regularly in pre-determined workgroups to discuss progress and continue practice-based learning though group critical appraisals of research evidence. - Participants from public health units met regularly with mentors to address emerging questions and strategize organization change for evidence-informed decision-making. - At the conclusion of the workshop series, participants completed a rapid review. | 7 |  |
|  | **Who provided**  Four knowledge translation specialists with graduate degrees and experience in evidence-informed public health delivered workshop lectures and acted as program mentors. | 6 |  |
|  | **How**   - Participants were recruited through the NCCMT’s newsletter and through follow-up contact to organizations with previously expressed interest in the Program. - Workshops were conducted in person, face-to-face; large group lectures were followed by small group-based sessions. - Monthly working groups sessions were held virtually, face-to-face. - Public health mentor meetings were conducted via tele-conferencing. | 6  7  7  7 |  |
|  | **Where**  McMaster University, Hamilton, ON | 7 |  |
|  | **When and how much**  Participants took part in 11 face-to-face sessions (5 days at start, 3 days at 6 months and 2 days at 12 months); each session taking place from 9AM – 4 PM. Participants from public health units participated in 12 additional 1-hour meetings. | 7 |  |
|  | **Tailoring**  The program began with an organizational assessment with the senior leadership team of each health unit. Assessments identified the leadership team’s EIDM priorities for the program specifically and the organization generally. | 7 |  |
|  | **Modifications**  None | N/A |  |
|  | **How well \| Planned**  Attendance at face-to-face sessions was tracked. | Figure 1 |  |
|  | **How well \| Actual**  Fifty-five participants took part in the program. Five participants left the program due to role changes within their public health unit or leaves of absence and were replaced by new participants. One participant was added to the program following the first workshop based on interest in the program. | Figure 1 |  |
