# Supplementary material for: A pragmatic evaluation of a public health knowledge broker mentoring education program: a convergent mixed methods study
Source: Implement Sci Commun. 2022 Feb 15;3:18. doi: 10.1186/s43058-022-00267-5 (PMC8845284; doi:10.1186/s43058-022-00267-5)
Supplement: Supplementary file 2 — Additional file 2: Appendix 2. Interview structure. [file 43058_2022_267_MOESM2_ESM.docx]

# Appendix 2

Interview Structure

Preamble: Thank you for agreeing to speak with me today. I would like to talk to you about what has happened around KB or EIDM in your organization since you (or your staff, or manager) took part in the KB mentoring initiative. With your permission, I would like to tape record our conversation which should last about 30 minutes. Only the transcriptionist and I will listen to the tape and then it will be destroyed. We will produce a written transcript or notes of our conversation. The information you provide will be collated with the responses from others involved in the KB Mentoring Initiative and included in one or more evaluation reports. Any excerpts from our conversation that are included in the reports will be presented so that they cannot be traced back to you or your organization.

Are you comfortable with taping our conversation?

1. What is your role in this organization or your job? Are you in the same role as when you took the training?
2. How many other people from your health unit took the training? How many are still with the health unit?
3. What is your connection to the KB Mentoring Initiative (participant, manager of participant, Director of participant, staff of participant)?
4. When did you take the training?
5. What impact has your involvement in the KB mentoring program had on you personally? On your organization? On KB or EIDM?
   1. Probe re: ability to do 7 steps
   2. Probe re: any impacts on organizational policy or processes?
   3. Probe re: can you point to something that is done differently because of the KB Mentoring?
6. What further engagement have you had with NCCMT since the KB mentoring program?
   1. To what extent did your involvement in KB Mentoring Initiative foster a change in your engagement with NCCMT? Please explain.
7. What was the value of KB Mentoring Initiative in your organization’s EIDM journey? (how KB initiative made a difference, e.g., provided tools, provided a process, provided an impetus, provided protected time, etc.)
8. Given what has happened since you were engaged in the initiative, do you have any suggestions for what could have been improved in the KB Mentoring Initiative?
9. What would further support EIDM in your organization?
10. How important has NCCMT’s support or resources been in furthering EIDM? What would have happened if they did not exist?
